# PD-L1 expression and its clinicopathological correlation in advanced esophageal squamous cell carcinoma in a Chinese population

**DOI:** 10.1186/s13000-019-0778-4

**Published:** 2019-01-26

**Authors:** Lulu Rong, Yong Liu, Zhouguang Hui, Zitong Zhao, Yueming Zhang, Bingzhi Wang, Yanling Yuan, Wenbin Li, Lei Guo, Jianming Ying, Yongmei Song, Luhua Wang, Zhongren Zhou, Liyan Xue, Ning Lu

**Affiliations:** 1 Department of Pathology and Resident Training Base, National Cancer Center/National Clinical Research Center for Cancer/Cancer Hospital, Chinese Academy of Medical Sciences and Peking Union Medical College, Beijing, 100021 China; 2 Department of Endoscopy, National Cancer Center/National Clinical Research Center for Cancer/Cancer Hospital, Chinese Academy of Medical Sciences and Peking Union Medical College, Beijing, 100021 China; 3 Department of Radiotherapy, National Cancer Center/National Clinical Research Center for Cancer/Cancer Hospital, Chinese Academy of Medical Sciences and Peking Union Medical College, Beijing, 100021 China; 4 State Key Laboratory of Molecular Oncology, National Cancer Center/National Clinical Research Center for Cancer/Cancer Hospital, Chinese Academy of Medical Sciences and Peking Union Medical College, Beijing, 100021 China; 5 Department of Pathology and Immunology, Washington University School of Medicine, St. Louis, USA; 6 Center for Cancer Precision Medicine, National Cancer Center/National Clinical Research Center for Cancer/Cancer Hospital, Chinese Academy of Medical Sciences and Peking Union Medical College, Beijing, 100021 China

**Keywords:** PD-L1, Esophageal squamous cell carcinoma, Clinicopathological parameters, Disease-free survival, Recurrence

## Abstract

**Background:**

Programmed death ligand 1 (PD-L1) is a ligand for the inhibitory programmed cell death protein 1 (PD-1), which are targeted by several anti-PD-1 and PD-L1 drugs for a variety of human cancers. However, only a few studies have evaluated PD-L1 expression in esophageal squamous cell carcinoma (ESCC) with a large Chinese cohort. Our present study is to evaluate the association of PD-L1 expression with clinicopathological features on ESCC.

**Methods:**

Using tissue microarray and immunohistochemistry, PD-L1 expression on tumor cells and tumor-infiltrating immune cells was studied in 378 advanced ESCC patients without neoadjuvant chemoradiotherapy. Its correlation with clinicopathological parameters was analyzed.

**Results:**

PD-L1 was expressed on 29.9% (113/378) ESCC tumor cells and 40.2% (152/378) tumor-infiltrating immune cells. PD-L1 expression in tumor cells was significantly correlated with age, degree of differentiation, T stage, N stage and metachronous hematogenous metastasis, and PD-L1 expression in tumor-infiltrating immune cells was significantly associated with N stage (*P* < 0.05). Patients with PD-L1 expression in tumor cells had poor disease-free survival (Hazard ratio [HR] = 1.436, *P* = 0.009). There was a positive association between tumor cells and tumor-infiltrating immune cells for PD-L1 expression (*r* = 0.16, *P* = 0.002). However, PD-L1 expression in tumor-infiltrating immune cells was not significantly correlated with disease-free survival and overall survival.

**Conclusions:**

PD-L1 expression in tumor cells and tumor infiltrating immune cells is not only an indicator for immunotherapy, but also significantly related with age, differentiation, stage, metastasis and disease free survival.

## Background

Esophageal squamous cell carcinoma (ESCC) is the 3rd most common cause of death from cancer in China [1]. In spite of great progress of surgery and other treatments, the prognosis of patients with advanced ESCC is still very poor [2]. New therapies are urgently needed to improve the survival rate and survival quality for advanced ESCC.

The immune checkpoint programmed cell death protein 1 (PD-1) is expressed in tumor-infiltrating immune cells including T-lymphocytes, B-lymphocytes, natural killer cells, monocytes, and dendritic cells. It is engaged by the tumor expressed ligands including programmed death ligand 1 (PD-L1) and PD-L2, which increases the apoptosis of activated tumor-reactive T-cells and promotes the growth of tumor cells in vivo [3]. Recently, PD-L1 immune checkpoint inhibitor antibodies in multiple clinical trials were used to treat many cancer types [4–9], including melanoma, non-small cell lung cancer (NSCLC), hepatocellular carcinoma, esophageal cancer and bladder cancer.

In recent years, the relationship between PD-L1 expression and clinical outcomes have been studied in ESCC [10–16]. However, the association of PD-L1 expression with the clinicopathological relationship in ESCC remains controversial. Some studies demonstrated that PD-L1 expression was correlated with poor prognosis [10, 17], while some studies suggested that PD-L1 could be a favorable prognostic indicator in ESCC [12]. In one study, PD-L1 expression was found to be not related to prognosis [18]. Our study aims to study the expression of PD-L1 in T2-T4a ESCC in a Chinese population and analyze its correlation with clinicopathological parameters and prognosis. These might provide a clue of the potential immune based therapy strategy for ESCC patients.

## Methods

### Tissue samples

A total of 378 patients with primary esophageal squamous cell carcinoma, who received radical esophagectomy without neoadjuvant chemoradiotherapy in National Cancer Center/National Clinical Research Center for Cancer/Chinese Academy of Medical Sciences and Peking Union Medical College between April 1999 and March 2003, were included in this retrospective study. This study contained T2 stage (*n* = 103), T3 stage (*n* = 238), T4a stage (*n* = 37), N1 stage (*n* = 116), N2 stage (*n* = 52), and N3 stage (*n* = 21) cases. These cases were non-consecutive cases which had complete follow-up data. The patients’ medical records were reviewed to obtain patients’ clinicopathological parameters, including age at diagnosis, gender, tumor differentiation, tumor location, vascular invasion, perineural invasion, metachronous hematogenous metastasis, and pathological TNM stage (Table 2). The HE slides were reviewed by two pathologists (Lulu Rong and Liyan Xue) to obtain pathological parameters, and any arguments were resolved by consensus review. TNM staging according to the 8th American Joint Committee on Cancer (AJCC) TNM classification [19].

### Tissue microarray construction

All tumor samples were fixed in 10% neutral buffered formalin for 12–48 h and embedded in paraffin. Tissue microarrays (TMAs) were constructed from three 0.6-mm cores of tumor tissue and three 0.6-mm cores of normal epithelium from each case using a Manual Tissue Arrayer (MTA-1, Beecher Instruments, Silver Spring, MD).

### Immunohistochemistry (IHC)

The IHC Envision staining method was used for immunohistochemical staining. Antigen retrieval was performed by pressure oven 2.5 min in EDTA (pH 9.0). Slides were incubated for 15 min with H_2_O_2_ (Dako, Glostrup, Denmark). The primary antibody, rabbit anti-PD-L1 monoclonal antibody (clone SP142; 1:50, Spring Bioscience, Pleasanton, CA, USA) was incubated sequentially for two hours at room temperature. The bound antibody was then detected with the horseradish peroxidase (HRP)-labeled goat anti-mouse/rabbit secondary antibody (Dako, Glostrup, Denmark) for 30 min at room temperature. Finally, reaction products were visualized with 3, 3′-diaminobenzidine (DAB, Dako, Glostrup, Denmark). Slides were lightly counterstained with hematoxylin.

### Evaluation of IHC

In our study, tumor cells and tumor-infiltrating immune cells were quantified by evaluating the percentage of stained and unstained cells (number of PD-L1-positive tumor cells/number of all tumor cells or number of PD-L1-positive tumor-infiltrating immune cells/number of all tumor-infiltrating immune cells). The proportion of PD-L1 positive expression in tumor cells was estimated as different thresholds (< 1%, 1–9%, 10–49%, or ≥ 50%) (Table 1), Tumor cells were designated PD-L1 positive expression when ≥1% of the tumor cells were positive for PD-L1, evaluated as partially or completely staining on the cell membrane or cell membrane and cytoplasm for tumor cells. Positive PD-L1 expression on immune cells was defined as ≥1% positive cells with membranous or cytoplasmic staining. Necrotic areas were excluded from scoring. IHC results were evaluated by two pathologists (Lulu Rong and Liyan Xue) in a blinded manner. Doubtful cases were discussed by the two pathologists using a multiheaded microscope until consensus was achieved.

**Table 1:** PD-L1 expression based on the percent-positive tumor cells by immunohistochemical staining

PD-L1 expression in tumor cells (%)	< 1%	1–9%	10–49%	≥50%
Overall	265(70.1%)	51(13.5%)	34(9.0%)	28(7.4%)

### Statistical analysis

SPSS 19.0 software was used for statistical analysis. The significance of the difference between PD-L1 expression and clinicopathological parameters was assessed by the univariate Logistic regression analysis. The relationship between tumor cells and tumor-infiltrating immune cells for PD-L1 expression was examined using correlation analysis. Disease-free survival rates and overall survival rates were calculated and survival curves were constructed using the Kaplan-Meier method, and the log-rank test was used to evaluate the statistical significance of differences. The prognostic significance of clinicopathological parameters was determined using univariate Cox proportional hazards analysis. To assess the presence of possible confounding variables, the backward stepwise multivariate Cox proportional hazards analysis was applied for factors that achieved significance in univariate Cox proportional hazards analysis. Hazard ratios (HR) with 95% confidence intervals (CIs) were reported.

All statistical tests were conducted as two-sided, and *P* < 0.05 was considered to indicate a statistically significant difference.

## Results

### PD-L1 expression in ESCC tumor cells and its correlation with clinicopathological parameters

**Fig. 1:**
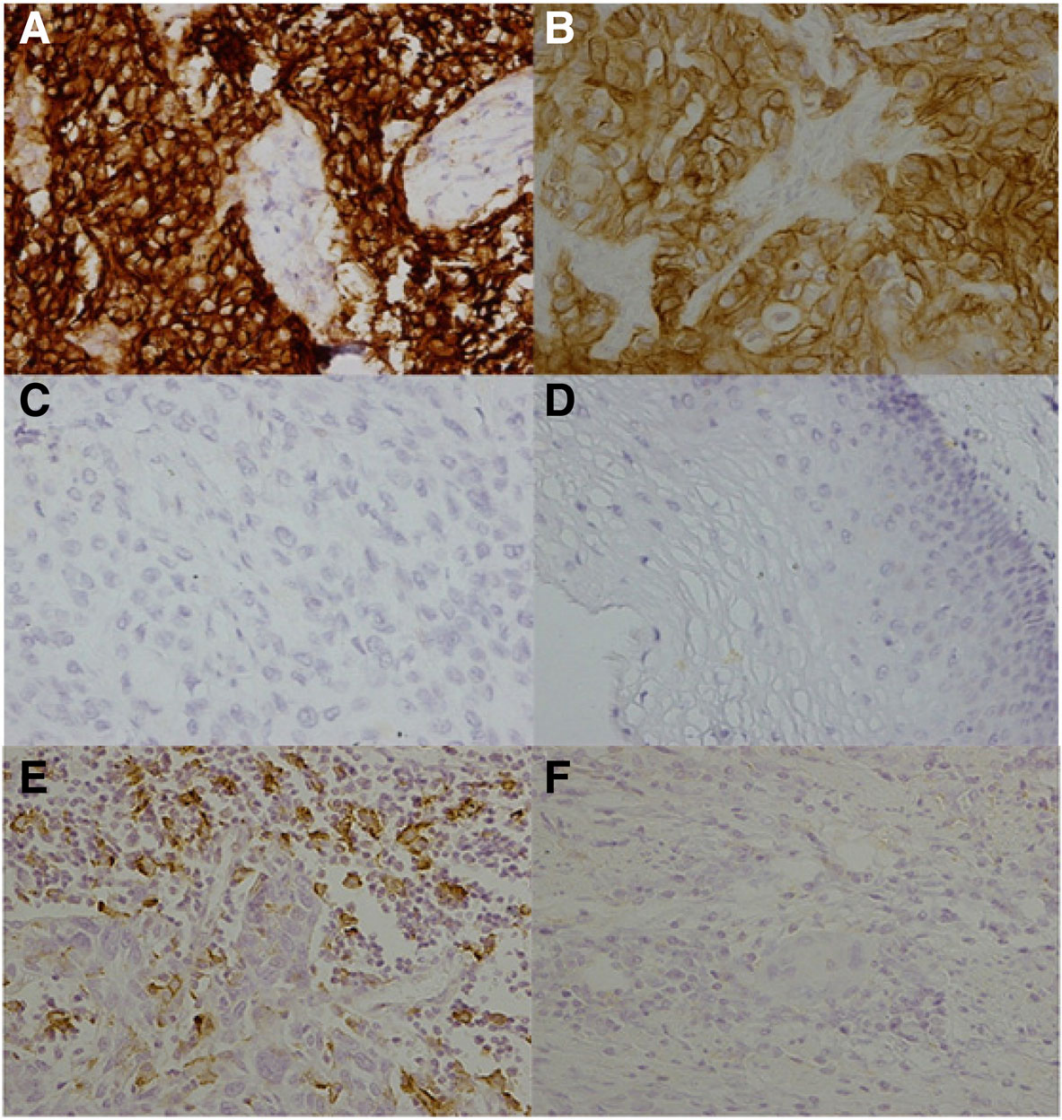
PD-L1 expression in esophageal squamous cell carcinoma (ESCC) and adjacent non-malignant epithelium by immunohistochemistry staining. **a** Strong positive expression of PD-L1 in tumor cells. Original magnification, 400×. **b** Weak positive expression of PD-L1 in tumor cells. Original magnification, 400×. **c** Negative expression of PD-L1 in tumor cells. Original magnification, 400×. **d** The adjacent non-malignant epithelium. Original magnification, 200×. **e** Positive expression of PD-L1 in tumor-infiltrating immune cells. Original magnification, 400×. **f** Negative expression of PD-L1 in tumor-infiltrating immune cells. Original magnification, 400 ×

PD-L1 was found to be located on the cell membrane and/or cytoplasm in ESCC tumor cells and tumor-infiltrating immune cells (Fig. 1). The expression of PD-L1 in ESCC tumor cells was positive in 29.9% (113/378), which was associated with various clinicopathological parameters including age, degree of differentiation, T stage, N stage and metachronous hematogenous metastasis. Poor differentiation ESCC had higher PD-L1 positive expression (Table 2).

**Table 2:** Relationships between clinicopathological characteristics and PD-L1 expression in tumor cells

Clinicopathological characteristics	Overall	PD-L1 (+)	PD-L1 (−)	Odds ratio (95% CI)	Global P
	378	113(29.9%)	265(70.1%)		
Age at diagnosis				1.563(1.003–2.438)	0.049
≥ 60 years	188	65(34.6%)	123(65.4%)		
< 60 years	190	48(25.3%)	142(74.7%)		
Gender				0.800(0.461–1.386)	0.425
Male	307	89(29.0%)	218(71.0%)		
Female	71	24(33.8%)	47(66.2%)		
Tumor differentiation					
Well	85	21(24.7%)	64(75.3%)	1	
Moderate	190	51(26.8%)	139(73.2%)	1.118(0.621–2.013)	0.710
Poor	91	38(41.8%)	53(58.2%)	2.185(1.146–4.166)	0.018
Basaloid	12	3(25.0%)	9(75.0%)	1.016(0.251–4.105)	0.982
Location					
Upper thoracic	61	18(29.5%)	43(70.5%)	1	
Middle thoracic	209	59(28.2%)	150(71.8%)	0.940(0.502–1.759)	0.846
Lower thoracic	108	36(33.3%)	72(66.7%)	1.194(0.605–2.358)	0.609
PT status				1.821(1.068–3.106)	0.028
pT2	103	22(21.4%)	81(78.6%)		
pT3-4a	275	91(33.1%)	184(66.9%)		
PN status				1.541(1.210–1.963)	< 0.001
pN0	189	40(21.2%)	149(78.8%)		
pN1	116	43(37.1%)	73(62.9%)		
pN2	52	20(38.5%)	32(61.5%)		
pN3	21	10(47.6%)	11(52.4%)		
Vascular invasion				1.025(0.656–1.600)	0.915
No	219	65(29.7%)	154(70.3%)		
Yes	159	48(30.2%)	111(69.8%)		
Perineural invasion				0.823(0.512–1.324)	0.422
No	253	79(31.2%)	174(68.8%)		
Yes	125	34(27.2%)	91(72.8%)		
Metachronous hematogenous metastasis				2.030(1.171–3.520)	0.012
No	313	85(27.2%)	228(72.8%)		
Yes	65	28(43.1%)	37(56.9%)		

### PD-L1 expression in ESCC tumor-infiltrating immune cells and its correlation with clinicopathological parameters

**Fig. 2:**
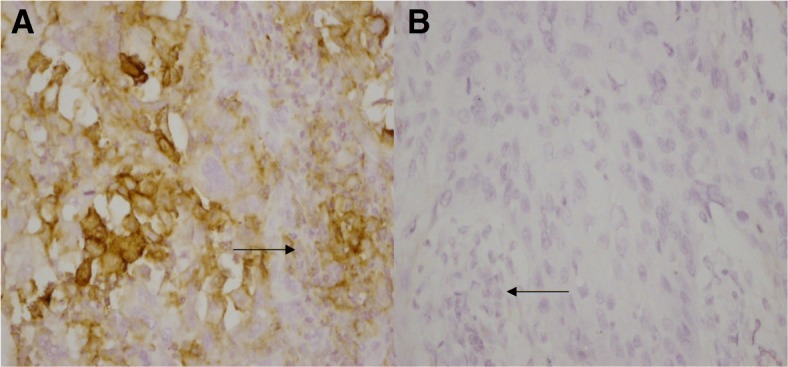
PD-L1 expression in esophageal squamous cell carcinoma (ESCC) tumor cells and tumor-infiltrating immune cells. **a** PD-L1 positive expression in tumor cells and tumor-infiltrating immune cells. Original magnification, 400×, (Black arrow shows the tumor-infiltrating immune cells). **b** PD-L1 negative expression in tumor cells and tumor-infiltrating immune cells. Original magnification, 400×, (Black arrow shows the tumor-infiltrating immune cells)

PD-L1 positive expression in ESCC tumor-infiltrating immune cells was 40.2% (152/378) (Table 3). PD-L1 expression in tumor-infiltrating immune cells was significantly associated with N stage (*P* < 0.05) (Table 3). PD-L1 expression in tumor-infiltrating immune cells was significantly associated with PD-L1 expression in tumor cells (*r* = 0.16, *P* = 0.002; Table 4, Fig. 2).

**Table 3:** Relationships between clinicopathological characteristics and PD-L1 expression in tumor-infiltrating immune cells

Clinicopathological characteristics	Overall	PD-L1 (+)	PD-L1 (−)	Odds ratio (95% CI)	Global P
	378	152(40.2%)	226(59.8%)		
Age at diagnosis				0.853(0.565–1.288)	0.450
≥ 60 years	188	72(38.3%)	116(61.7%)		
< 60 years	190	80(42.1%)	110(57.9%)		
Gender				1.205(0.707–2.055)	0.494
Male	307	126(41.0%)	181(59.0%)		
Female	71	26(36.6%)	45(63.4%)		
Tumor differentiation					
Well	85	32(37.6%)	53(62.4%)	1	
Moderate	190	77(40.5%)	113(59.5%)	1.129(0.667–1.909)	0.652
Poor	91	37(40.7%)	54(59.3%)	1.135(0.619–2.081)	0.683
Basaloid	12	6(50.0%)	6(50.0%)	1.656(0.492–5.575)	0.415
Location					
Upper thoracic	61	24(39.3%)	37(60.7%)	1	
Middle thoracic	209	71(34.0%)	138(66.0%)	0.793(0.441–1.428)	0.440
Lower thoracic	108	57(52.8%)	51(47.2%)	1.723(0.911–3.260)	0.094
PT status				1.211(0.760–1.931)	0.421
pT2	103	38(36.9%)	65(63.1%)		
pT3-4a	275	114(41.5%)	161(58.5%)		
PN status				1.630(1.286–2.067)	< 0.001
pN0	189	53(28.0%)	136(72.0%)		
pN1	116	61(52.6%)	55(47.4%)		
pN2	52	26(50.0%)	26(50.0%)		
pN3	21	12(57.1%)	9(42.9%)		
Vascular invasion				0.729(0.479–1.110)	0.141
No	219	95(43.4%)	124(56.6%)		
Yes	159	57(35.8%)	102(64.2%)		
Perineural invasion				0.939(0.606–1.455)	0.778
No	253	103(40.7%)	150(59.3%)		
Yes	125	49(39.2%)	76(60.8%)		
Metachronous hematogenous metastasis				0.989(0.574–1.707)	0.969
No	313	126(40.3%)	187(59.7%)		
Yes	65	26(40.0%)	39(60.0%)

**Table 4:** The relationship between tumor cells and tumor-infiltrating immune cells for PD-L1 expression

Variables	Overall	Tumor cells PD-L1(−)	Tumor cells PD-L1(+)	r	*P* value
Tumor-infiltrating immune cells PD-L1(−)	226	172(76.1%)	54(23.9%)	0.160	0.002
Tumor-infiltrating immune cells PD-L1(+)	152	93(61.2%)	59(38.8%)

### Correlation between PD-L1 expression in ESCC tumor cells and prognosis

**Fig. 3:**
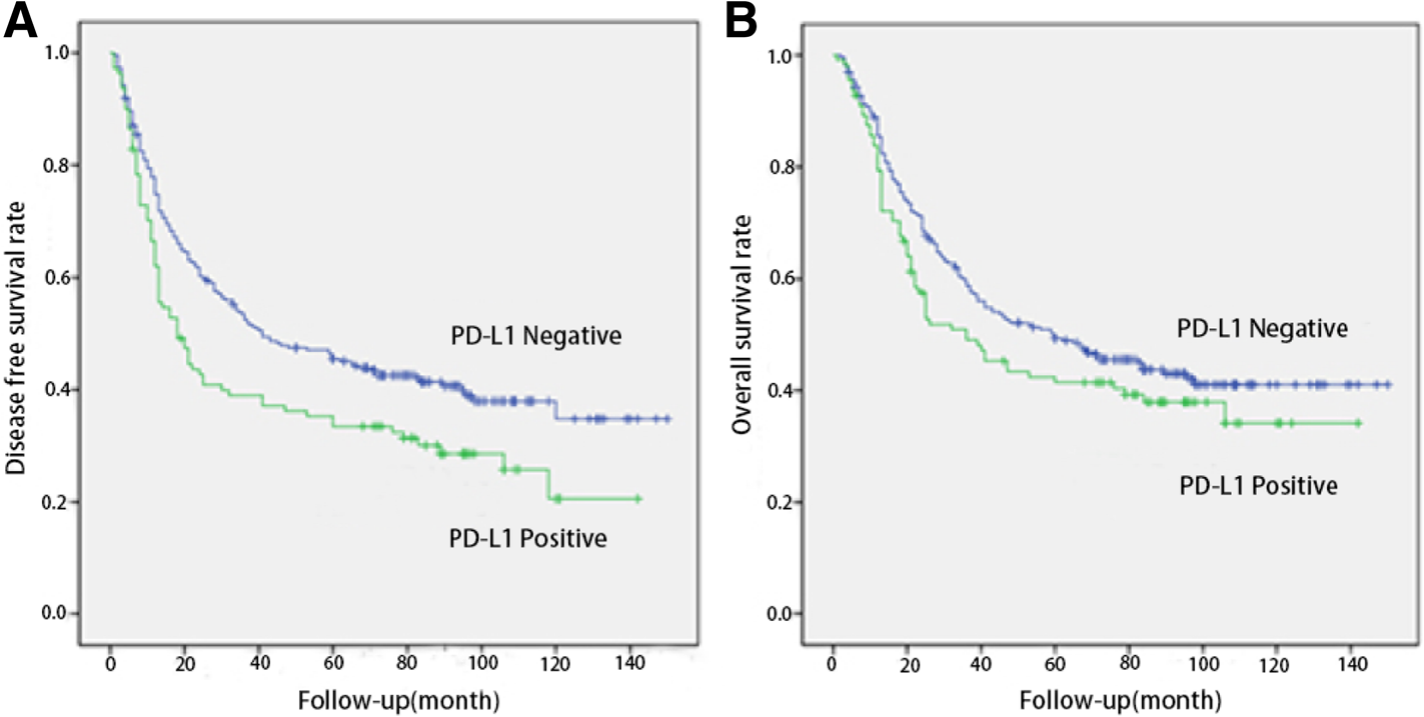
Kaplan-Meier curves of disease-free survival (DFS) and overall survival (OS) in esophageal squamous cell carcinoma (ESCC) based upon PD-L1 expression in tumor cells. **a** Patients with PD-L1 expression had significantly shorter DFS than those without PD-L1 expression (median DFS time: 18 verse 41 months, *P* = 0.008). **b** There was no statistically significant difference in OS between the patients with positive and negative PD-L1 staining (median OS time: 36 verse 60 months, *P* = 0.140)

The median disease-free survival time (DFS) was 41 months in PD-L1 negative patients and 18 months in PD-L1 positive patients, respectively. PD-L1 expression was significantly associated with shorter DFS (*P* = 0.008). The median overall survival time (OS) was 60 months in PD-L1 negative patients and 36 months in PD-L1 positive patients, respectively. PD-L1 expression patients had a shorter OS, but not statistically significant (*P* = 0.140) (Fig. 3). Univariate Cox analysis showed that patients with PD-L1 expression had poor DFS (Hazard ratio [HR] = 1.436, 95% CI: 1.095–1.883, *P* = 0.009) (Table 5). However, multivariate Cox analysis failed to show PD-L1 as an independent prognostic factor (Table 6).

**Table 5:** Univariate COX analysis to determine factors associated with patient survival for PD-L1 expression in tumor cells

Variables	Overall survival			Disease-free survival		
	Hazard ratios	95%CI	*P* value	Hazard ratios	95%CI	*P* value
PD-L1						
Negative	1			1		
Positive	1.240	0.929-1.653	0.144	1.436	1.095-1.883	0.009
Age						
<60 years	1			1		
≥60 years	1.303	0.995-1.705	0.054	1.205	0.932-1.557	0.154
Gender						
Female	1			1		
Male	1.033	0.737-1.448	0.849	0.942	0.684-1.297	0.714
Tumor differentiation						
Well	1			1		
Moderate	1.383	0.954-2.005	0.087	1.458	1.025-2.074	0.036
Poor	2.150	1.435-3.222	<0.001	2.180	1.478-3.216	<0.001
Basaloid	1.774	0.828-3.804	0.141	2.162	1.052-4.444	0.036
Location						
Upper thoracic	1			1		
Middle thoracic	0.872	0.602-1.262	0.468	0.960	0.669-1.379	0.826
Lower thoracic	0.798	0.529-1.204	0.282	0.900	0.604-1.341	0.604
PT status						
pT2	1			1		
pT3-4a	1.348	0.984-1.847	0.063	1.377	1.019-1.859	0.037
PN status						
pN0	1			1		
pN1	2.175	1.575-3.002	<0.001	2.277	1.681-3.084	<0.001
pN2	3.703	2.542-5.394	<0.001	3.291	2.284-4.744	<0.001
pN3	9.401	5.707-15.486	<0.001	8.029	4.875-13.225	<0.001
Vascular invasion						
No	1			1		
Yes	1.853	1.415-2.427	<0.001	1.714	1.326-2.218	<0.001
Perineural invasion						
No	1			1		
Yes	1.707	1.297-2.245	<0.001	1.645	1.267-2.138	<0.001
Metachronous hematogenous metastasis						
No	1			1		
Yes	2.533	1.868-3.435	<0.001	3.638	2.716-4.873	<0.001

**Table 6:** Multivariate Cox analysis to determine independent factors associated with patient survival for PD-L1 expression in tumor cells

Variables	Overall survival			Disease-free survival		
	Hazard ratios	95%CI	*P* value	Hazard ratios	95%CI	*P* value
PD-L1						
Negative	1			1		
Positive	0.882	0.648-1.200	0.423	1.047	0.782-1.401	0.758
Age						
<60 years	1			1		
≥60 years	1.623	1.223-2.152	0.001	1.442	1.109-1.875	0.006
Tumor differentiation						
Well	1			1		
Moderate	1.081	0.736-1.586	0.691	1.192	0.834-1.703	0.334
Poor	1.369	0.890-2.107	0.153	1.533	1.030-2.280	0.035
Basaloid	1.109	0.507-2.425	0.796	1.128	0.538-2.367	0.750
PT status						
pT2	1			1		
pT3-4a	1.119	0.800-1.565	0.512	1.295	0.945-1.776	0.108
PN status						
pN0	1			1		
pN1	2.054	1.485-2.841	<0.001	2.194	1.617-2.977	<0.001
pN2	3.316	2.242-4.906	<0.001	3.188	2.201-4.616	<0.001
pN3	8.016	4.726-13.749	<0.001	7.865	4.682-13.210	<0.001
Vascular invasion						
No	1			1		
Yes	1.291	0.970-1.718	0.080	1.103	0.831-1.464	0.497
Perineural invasion						
No	1			1		
Yes	1.328	0.999-1.764	0.051	1.325	1.012-1.736	0.041
Metachronous hematogenous metastasis						
No	1			1		
Yes	2.073	1.520-2.827	<0.001	3.359	2.499-4.517	<0.001

### Correlation between PD-L1 expression in ESCC tumor-infiltrating immune cells and prognosis

The median DFS was 36 months in PD-L1 positive tumor-infiltrating immune cells patients and 34 months in PD-L1 negative patients, respectively. The median OS was 53 months in PD-L1 positive tumor-infiltrating immune cells patients and 47 months in PD-L1 negative patients, respectively. No statistical significance was found in both DFS and OS between PD-L1 positive and negative tumor-infiltrating immune cell patients (median OS, 53 versus 47 months, *P* = 0.901; and median DFS, 36 versus 34 months, *P* = 0.706).

## Discussion

Our study is very unique compared to other reports since we selected the ESCC esophagectomy samples without neoadjuvant chemoradiotherapy, which excluded the possible treatment effect on PD-L1 expression. In the current study, we found that 29.9% of T2-T4a ESCC cases were positive for PD-L1 in tumor cells and 40.2% positive in tumor-infiltrating immune cells. In addition, PD-L1 expression in ESCC tumor cells was associated with various clinicopathological parameters including age, degree of differentiation, stage, metastasis and DFS.

PD-L1 positive expression in ESCC tumor cells has been reported in several studies from 18.9 to 45% [10–14]. Our current study showed that 29.9% of ESCC cases were positive for PD-L1 in tumor cells. These differences might be due to several factors including antibodies, cut-off points, neoadjuvant therapy or IHC methods. For example, Chen and his colleagues found that 45% of ESCC tissues showed positive PD-L1 immunoreactivity [10]. However, their study included neoadjuvant chemoradiotherapy patients. Based on the data from another study, Lim et al. found PD-L1 (5H1) expression increased in ESCC patients who received neoadjuvant therapy [11]. Our present study excluded the patients who had accepted neoadjuvant chemoradiotherapy. In addition, Ito S et al. found that 18.9% of ESCC tissues had positive PD-L1 (LS-B480) expression [13]. However, their study used the scoring for PD-L1 expression based on adding both the proportion score and the intensity score with cut-off as ≥7, which is different from the current PD-L1 evaluation guideline from clinical application. In our study, we designated PD-L1 positive when ≥1% of the tumor cells or immune cells were positive for PD-L1.

The association between PD-L1 expression and clinicopathological features was reported in several studies. The lymph node metastasis and tumor stages were found to associate with PD-L1 expression in most studies [10–13]. In our study, we had similar finding. In addition, we also showed that PD-L1 expression was associated with age and tumor differentiation. We found the PD-L1 expression were significantly higher in old patients (35%) than young patients (25%). We also found that poor differentiation ESCC had higher PD-L1 expression (42%) compared to well (25%) and moderate (27%) differentiation groups. We did not find that tumor location was associated with PD-L1 expression, which was reported by Chen’s study [10].

The association of PD-L1 expression with ESCC patient’s prognosis was controversial. Most of studies found that PD-L1 expression was significantly related with worse overall survival or disease free survival [10, 11, 13–17, 20, 21]. However, a few studies reported that PD-L1 positivity was associated with a favorable prognosis [12, 22, 23]. In our study, we found that PD-L1 expression in tumor cells was significantly correlated with DFS (41 months vs 18 months, PD-L1 negative vs positive) with univariate Cox analysis, but multivariate Cox analysis failed to show PD-L1 as an independent prognostic factor. In addition, we found that the median OS was 60 months in PD-L1 negative patients and 36 months in PD-L1 positive patients, respectively. However, it was not statistically significant (*P* = 0.140). Based on current data, PD-L1 expression might be related with poorer prognosis, which might be caused by the association of PD-L1 expression with elder patients, lymph node metastasis, poor differentiation and later stages.

Furthermore, we found the PD-L1 expression in ESCC tumor-infiltrating immune cells was 40.2% (152/378). PD-L1 expression in tumor-infiltrating immune cells was significantly associated with N stage and PD-L1 expression in tumor cells. We analyzed the prognostic relevance of PD-L1 expression in tumor-infiltrating immune cells and showed that the median OS and DFS were longer in patients with PD-L1 expression in tumor-infiltrating immune cells, which was consistent with recent study by Zhang et al. [18]. This might be an indicator of a host immune response to tumor cells that led to improve survival.

In addition, we also evaluated PD-L1 expression if the cut-off point was 10% or 50%, based on the percent-positive tumor cells by IHC, the PD-L1 positive expression in tumor cells was 16.4 and 7.4%, respectively. The positive expression of PD-L1 in ESCC tumor cells was associated with tumor differentiation, T stage and shorter DFS (Hazard ratio [HR] = 1.488, *P* = 0.017) when the cut-off point was 10%, but not correlated with OS (Hazard ratio [HR] = 1.255, *P* = 0.210), just as the result of the cut-off point was 1% (OS, Hazard ratio [HR] = 1.240, *P* = 0.144; and DFS, Hazard ratio [HR] = 1.436, *P* = 0.009). If the cut-off point was 50%, PD-L1 expression was just associated with age, but no significant association was found between PD-L1 expression and the prognosis (OS, Hazard ratio [HR] = 1.304, *P* = 0.293; and DFS, Hazard ratio [HR] = 1.344, *P* = 0.218).

PD-L1 positive expression in our data was relative lower than the results from one meta-analysis [24]. The lower PD-L1 expression in our TMA data might be caused by tumor heterogeneity [25]. In order to reduce the discordance of PD-L1 expression, TMAs in our study were constructed from three cores of tumor tissue and three cores of normal epithelium from each case. One study showed that TMAs correlated moderately well with that in the corresponding whole slide surgical specimens [26].

To our knowledge, this is the first study that systematically analyzed the prognostic relevance of PD-L1 expression in tumor cells and tumor-infiltrating immune cells in advanced ESCC patients who received radical esophagectomy without neoadjuvant chemoradiotherapy. ESCC patients with T2 or more advanced stages had worse prognosis than T1 stage and were more likely to be benefit from immunotherapy. The T1 stage ESCC patients had good prognosis, most of whom needed no adjuvant therapy after surgery, so we didn’t include the T1 stage ESCC patients in our study. However, our study had some limitations. We examined PD-L1 expression mainly in only sampled a small esophageal volume to examine the prognostic value, which might result in undersampling of esophageal cancer tissue and it was a retrospective analysis of a population with different stages from a single institution, the issue should be best answered in context of a prospective study in a more patient population.

## Conclusions

In conclusion, PD-L1 expression in ESCC is not only an indicator for immunotherapy, but also is a potential prognostic marker for untreated ESCC patients. PD-L1 expression in both tumor cells and tumor-infiltrating immune cells is significantly associated multiple clinicopathological features including age, differentiation, stage and metastasis.

## Abbreviations

AJCC: American Joint Committee on Cancer
DFS: Disease-free survival
ESCC: Esophageal squamous cell carcinoma
FDA: US Food and Drug Administration
IHC: Immunohistochemistry
NSCLC: Non-small cell lung cancer
OS: Overall survival
PD-L1: Programmed cell death ligand 1
TMAs: Tissue microarrays
